# Supplementary material for: Cerebrovascular Disease Hospitalization Rates in End-Stage Kidney Disease Patients with Kidney Transplant and Peripheral Vascular Disease: Analysis Using the National Inpatient Sample (2005–2019)
Source: Healthcare (Basel). 2024 Feb 10;12(4):454. doi: 10.3390/healthcare12040454 (PMC10887507; doi:10.3390/healthcare12040454)
Supplement: Supplementary file 1 [file healthcare-12-00454-s001.zip › healthcare-2809985-supplementary.pdf]

Supplemental Table S1: Key Study Variables

Category	Variable	Variable Description
**Dependent Variables**	Cerebral infarction due to thrombosis, embolism, occlusion, and stenosis<br>Artery occlusion and stenosis resulting in cereral Ischemia<br>Intraoperative and Postprocedural Infarction<br>Non-traumatic Intracranial hemorrhage	Binary, indicating if encounter is identified with corresponding ICD-10/9 codes.
**Key Diagnoses of Interest**	End-Stage Renal Disease<br>Kidney Transplant<br>Peripheral Vascular Disease	Binary, indicating if encounter is identified with corresponding ICD-10/9 codes
**Comorbidity Index**	Elixhauser	Standard comorbidity index which utilizes ICD diagnosis codes to sort patient morbidities (Elixhauser et al., 1998).
**Sociodemographic Factors**	Age	Age in years at admission
	Race	Race/Ethnicity of patient
	Primary Expected Payer	Medicare, Medicaid, Private insurance, Self-pay, No charge, Other
	Patient's ZIP Code	Quartile categorization based on annually collected demographic data.
	Patient Location: NCHS Urban-Rural Code	Six-category urban-rural classification for U.S. counties developed by the National Center for Health Statistics (Data Access - Urban Rural Classification Scheme for Counties, 2022).

Supplemental Table S1. Key Study Variables.

Supplemental Table S2: ICD-10 and ICD-9 Descriptions

Diagnosis	ICD-10	ICD-9
End-Stage Renal Disease	N18.6	585.6
Kidney Transplant	Z94.0, Z48.22	556.9, V42.0
Peripheral Vascular Diseases	I73.8 I73.89 I73.9	443.89
Nontraumatic intracranial hemorrhage	I60.0 I60.00 I60.01 I60.02 I60.1 I60.10 I60.11 I60.12 I60.2 I60.3 I60.30 I60.31 I60.32 I60.4 I60.5 I60.50 I60.51 I60.52 I60.6 I60.7 I60.8 I60.9 I61.0 I61.1 I61.2 I61.3 I61.4 I61.5 I61.6 I61.8 I61.9 I62.0 I62.00 I62.01 I62.02 I62.03 I62.1 I62.9	430 431 432.1 432.0 432.9
Cerebral infarction due to thrombosis, embolism, occlusion, and stenosis	I63.0 I63.00 I63.01 I63.011 I63.012 I63.013 I63.019 I63.02 I63.03 I63.031 I63.032 I63.033 I63.039 I63.09 I63.1 I63.10 I63.11 I63.111 I63.112 I63.113 I63.119 I63.12 I63.13 I63.131 I63.132 I63.133 I63.139 I63.19 I63.2 I63.20 I63.21 I63.211 I63.212 I63.213 I63.219 I63.23 I63.231 I63.232 I63.233 I63.239 I63.29	433.91 433.21 433.01 433.11 433.81 433.91
Artery occlusion and stenosis resulting in cerebral ischemia	I65.0 I65.01 I65.02 I65.03 I65.09 I65.1 I65.2 I65.21 I65.22 I65.23 I65.29 I65.8 I65.9	433.20 433.00 433.10 433.80 433.90

Supplemental Table S2. Describes ICD-9 and ICD-10 codes which correspond to each condition used in the analysis.
